# Supplementary material for: Insights into a Cancer-Target Demethylase: Substrate Prediction through Systematic Specificity Analysis for KDM3A
Source: Biomolecules. 2022 Apr 27;12(5):641. doi: 10.3390/biom12050641 (PMC9139010; doi:10.3390/biom12050641)
Supplement: Supplementary file 1 [file biomolecules-12-00641-s001.zip › biomolecules-1664957-supp-for proof.pdf]

biomolecules

MDPI

Insights into a cancer-target demethylase: substrate prediction through systematic specificity analysis for KDM3A

Anand Chopra [1,2], William G. Willmore [1,2,*] and Kyle K. Biggar [1,2,*]

[1]Institute of Biochemistry, Carleton University, Ottawa, ON, Canada
[2]Department of Biology, Carleton University, Ottawa, ON, Canada
*Correspondence: KKB (kyle.biggar@carleton.ca) and WGW (bill.willmore@carleton.ca)

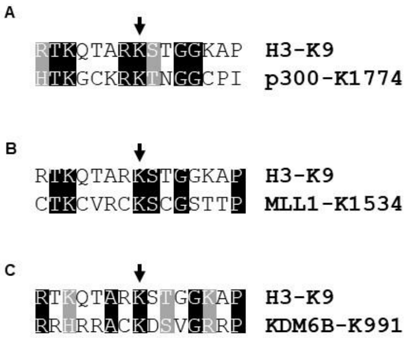

Supplementary Figure S1. Sequence alignment of the H3-K9 peptide with the newly identified *in vitro* substrates (**A**, p300-K1774; **B**, MLL1-K1534; **C**, KDM6B-K991) in Figure 4B. Black bars indicate exact residue match and gray bars indicate biochemical conservation. The location of the demethylation site is indicated by an arrow.

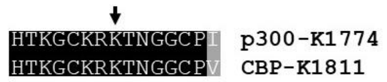

Supplementary Figure S2. Sequence alignment of the p300-K1774 15-mer peptide with a homologous region on CBP. Black bars indicate exact residue match and gray bars indicate biochemical conservation. The location of the demethylation site is indicated by an arrow.

Supplementary Table S2. Top scoring K-centered windows within the KDM3A interactome. Scoring was performed with a low-stringency KDM3A recognition motif (i.e., 0.5 threshold). All windows scored ≥ 3σ's above the population mean. Note that the methylation status of these predicted modification sites are currently unknown.

Protein	UniProt ID	K-position	Scored Window	Score
H3	P68431	10*	QTARKSTGG	9
RS4X	P62701	259	RLAAKQSSG	8
KMT2A	Q03164	234	GVKIKITHG	7
KMT2A	Q03164	1534	CVRCKSCGS	7
HS71A<br>HS71B	P0DMV8<br>P0DMV9	500	GKANKITIT	7
HUWE1	Q7Z6Z7	1470	MTAIKRNGA	7
HUWE1	Q7Z6Z7	3745	NKKAKQTGR	7
KDM6B	O15054	991	RRACKDSVG	7
MRTFB	Q9ULH7	879	EEAIKQTRS	7
MYC	P01106	157	QAARKDSGS	7
RASF6	Q6ZTQ3	32	KKEEKMTMM	7

VIR	Q69YN4	576	HLAEKTSSL	7
EP300	Q09472	1774	GCKRKTNGG	7
ARI1A	O14497	2189	IAVQKGSIG	7
JAK2	O60674	73	IAASKACGI	7
HNRPF	P52597	167	ELAEKALGK	7
CAPZB	P47756	159	EVQEKSSGR	7

* Denoted as such due to presence of N-terminal methionine within databases. Traditionally referred to as the 9th residue within literature.

Supplementary Table S5. Tabular form of the protein-protein association network depicted in Figure 5. The type of interaction that was identified by STRING is shown in each column, where green check marks indicate the presence of this interaction and dashes indicate the interaction was not determined.

Interactor 1	Interactor 2	Experimentally Determined	Curated Database	Textmining	Co-expression	Gene Neighborhood
EP300	SIRT1	✓	✓	✓	✓	-
EP300	MYOD1	✓	✓	✓	-	-
EP300	KDM6B	✓	-	✓	✓	-
EP300	MDM2	✓	✓	✓	-	-
EP300	SMAD2	✓	✓	✓	✓	-
EP300	YY1	✓	✓	✓	✓	-
EP300	KMT2A	✓	✓	✓	✓	-
KDM6B	SIRT1	✓	-	✓	✓	✓
KDM6B	KMT2A	✓	-	✓	✓	-
KDM6B	SMAD2	✓	-	✓	-	-
KMT2A	SIRT1	✓	-	✓	✓	-
MDM2	SIRT1	✓	-	✓	✓	-
MDM2	SMAD2	-	-	✓	✓	-
MDM2	YY1	✓	✓	✓	-	-
MYOD1	SIRT1	✓	✓	✓	-	-
MYOD1	YY1	✓	-	✓	-	-
MYOD1	SMAD2	✓	-	✓	-	-
SIRT1	SMAD2	✓	-	✓	✓	-
SIRT1	YY1	✓	-	✓	✓	-
SMAD2	YY1	✓	-	✓	✓	-
